# Prediction of Pad Wear Profile and Simulation of Its Influence on Wafer Polishing

**DOI:** 10.3390/mi14091683

**Published:** 2023-08-28

**Authors:** Pengjie Zheng, Dewen Zhao, Xinchun Lu

**Affiliations:** State Key Laboratory of Tribology, Tsinghua University, Beijing 100084, China; zpj16@mails.tsinghua.edu.cn (P.Z.); zhaodw@tsinghua.edu.cn (D.Z.)

**Keywords:** pad wear profile, kinematic simulation, experimental verification, pad–wafer contact stress, static

## Abstract

As feature sizes decrease, an investigation of pad unevenness caused by pad conditioning and its influence on chemical mechanical polishing is necessary. We set up a kinematic model to predict the pad wear profile caused by only diamond disk conditioning and verify it. This model shows the influences of different kinematic parameters. To keep the pad surface planar during polishing or only conditioning, we can change the sweep mode and range of the conditioner arm. The kinematic model is suitable for the prediction of the pad wear profile without considering the influence of mechanical parameters. Furthermore, based on the pad wear profile obtained from a real industrial process, we set up a static model to preliminarily investigate the influence of pad unevenness on the pad–wafer contact stress. The pad–wafer contact status in this static model can be approximated as an instantaneous state in a dynamic model. The model shows that the existence of a retaining ring helps to improve the wafer edge profile, and that pad unevenness can cause stress concentration and increase the difficulty in multi-zone pressure control of the polishing head.

## 1. Introduction

Chemical mechanical polishing (CMP) is widely used to achieve global planarization in integrated circuit (IC) manufacturing [1]. In a typical 12-inch CMP platform, the polishing head carries and presses the wafer on the polishing pad, and they all rotate in the same direction but eccentrically. The polishing pad is glued on a platen. The polishing head may oscillate along the platen radius. The pressurized chamber inside the polishing head may have multiple concentric zones. The retaining ring (RR) inside the polishing head helps keep the wafer from slipping outside. The slurry is supplied through a slurry arm hanging over the platen. A diamond disk is fixed on a sweep arm to restore the pad surface asperity and achieve a steady pad surface topography in order to keep the CMP process constant from wafer to wafer (WTW) during the whole lifetime of the pad [2].

The polishing pad is one of the consumables, and its surface topography and roughness influence parameters of the process such as the material removal rate (MRR), defects, and non-uniformity (NU). As polishing proceeds, asperities on the pad surface are gradually worn down and the surface becomes glazed. So, conditioning is necessary to keep the MRR constant. Conditioning by the diamond disk can lead to various cut rates on different pad radii, which makes the pad wear profile uncertain [3].

Studies on the prediction of pad wear profiles commonly use kinematic models, which include kinematic parameters such as platform geometric parameters, rotation speed, sweep mode, sweep range, and so on [2,3,4,5,6,7]. The Preston equation, an empirical formula initially used in the polishing MRR calculation [8], was also introduced for the pad cut rate (PCR) in these studies. Meanwhile, some other studies focused on proposing new relational formulas based on static models of the diamond/disk–pad interactions [9,10]. The statistical methods for the final results are also different, with some considering the number of scratches of all diamonds on the same radius section of the polishing pad while ignoring the scratch speed [5,6], and others calculating the total scratch distance or the total disk partition sweep area of all diamonds by unit [2,3,4,7].

In nearly all studies of the pad–wafer contact stress, the polishing pad is regarded as an ideal flat surface, and the focus is on the influence of the polishing head, the pressure of different membrane zones, and the retaining ring [11,12,13]. Studies on the influence of the pad surface unevenness are rare. The reasons for this are: firstly, the polishing pad and the wafer form an ultra-thin structure in the mechanical model due to its thickness–diameter quantitative relationship, which requires high computational power; secondly, there is a motion relationship between the wafer and the polishing pad, and the mechanical model should be dynamic, even the polishing slurry needs to be considered, which all increase the construction difficulty.

In this study, we constructed a kinematic model based on parameters obtained from a real industrial CMP platform (Type Universal-300-Plus from Hwatsing Co., Ltd., Tianjin 300350, China). The Preston equation is still adopted, and we calculated the total scratch distance of all diamonds by unit. According to this model, we understand the relationship between the PCR and kinematic parameters. The experimental results show a good approximation to the simulated ones. Furthermore, we constructed a static model to preliminarily investigate how the pad surface unevenness influences the pad–wafer contact stress, and thus, the polishing effects.

## 2. Kinematic Model

**Figure 1:**
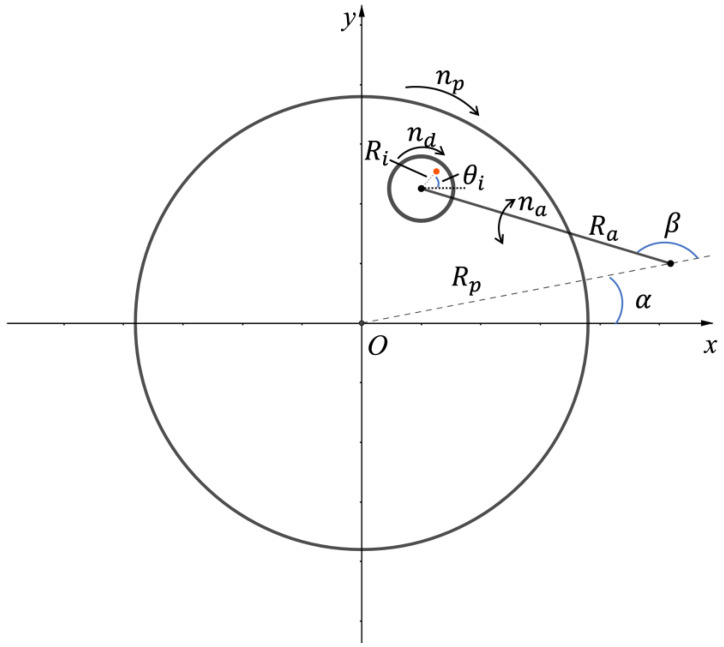
Schematic of the kinematic model and variables for the position of a single diamond.

Figure 1 shows the schematic of the kinematic model and variables for the position of a single diamond. Diamonds are simplified as points without size. Therefore, the diamonds’ height differences and their penetration difference or furrow cross influence will not be discussed here [14,15].

The Cartesian coordinate system is fixed on the pad without spinning. The arm center can be considered as spinning around the pad center in an inverse direction to the pad’s initial spinning direction.

We set the present total dressing time from the beginning as T (unit: second), the pad rotating speed as $n_{p}$ (unit: rotation per minute (RPM)), and the initial angle of the arm center to the pad center as $\alpha_{0}$ (unit: radian). The position equations of the arm center are as follows:

(1) $$\alpha \left(T\right)=\alpha_{0}-2\left(\frac{n_{p}}{60}\right)\pi T$$

(2) $$x_{ac}\left(T\right)=R_{p}cos(\alpha \left(T\right))$$

(3) $$y_{ac}\left(T\right)=R_{p}sin(\alpha \left(T\right))$$

The conditioner arm sweeps back and forth from the start point to the end point in some kind of sweep mode. We set the start angle of the disk center to the arm center as $\beta_{s}$ (unit: radian), the initial angle of the disk center to the arm center as $\beta_{0}$ (unit: radian), the sweep range of the conditioner arm as $\beta_{max}$ (unit: radian), and the arm sweep speed as $n_{a}$ (unit: RPM). Here is the equation for the sinusoidal sweep mode and the position equations of the disk center:

(4) $$\beta \left(T\right)=\beta_{s}-\frac{1}{2}\beta_{max}cos\left(arccos\left(\frac{2\left(\beta_{0}-\beta_{s}\right)}{\beta_{max}}\right)+2\left(\frac{n_{a}}{60}\right)\pi T\right)+\frac{1}{2}\beta_{max}$$

(5) $$x_{dc}\left(T\right)=x_{ac}\left(T\right)+R_{a}\cos(\alpha \left(T\right)+\beta \left(T\right))$$

(6) $$y_{dc}(T)=y_{ac}\left(T\right)+R_{a}\sin(\alpha \left(T\right)+\beta \left(T\right))$$

For a common diamond disk in a 12-inch-wafer CMP platform, diamond particles are electroplated or soldered onto stainless steel plates in many different designed or randomly distributed ways. We set the initial angle of the diamond to the disk center as $\theta_{i_{0}}$ (unit: radian), and the disk rotating speed as $n_{d}$ (unit: RPM). According to the assumption above, we can obtain the position equations of a single point, i.e., diamond i:

(7) $$\theta_{i}\left(T\right)=\theta_{i_{0}}+2\left(\frac{n_{d}}{60}\right)\pi T$$

(8) $$x_{i}\left(T\right)=x_{dc}\left(T\right)+R_{i}\cos(\alpha \left(T\right)+\beta \left(T\right)+\theta_{i}\left(T\right))$$

(9) $$y_{i}(T)=y_{dc}\left(T\right)+R_{i}\sin(\alpha \left(T\right)+\beta \left(T\right)+\theta_{i}\left(T\right))$$

**Figure 2:**
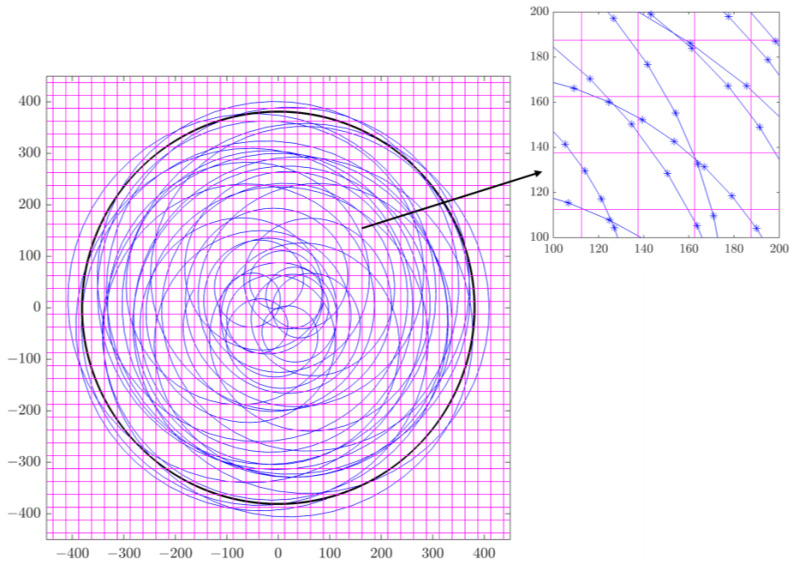
Trajectory of a single diamond (example) on the pad (20 s) and its distribution among all square units.

All of the above equations were input into the commercial software MATLAB (MathWorks, Version 9.12.0.2170939 (R2022a) Update 6, Natick, MA, U.S.), and we calculated the position of each diamond on the polishing pad and plotted its trajectory. Figure 2 shows the trajectory of an example diamond on the polishing pad for a total of 20 s and its distribution among all square units.

As mentioned above, here is the Preston equation:

(10) MRR=kPv

k is an empirical constant based on the experimental data, which varies under different conditions. P is the downforce/pressure of the polishing head, and v is the relative velocity between the wafer and pad.

Therefore, we can obtain the equation for the *PCR*:

(11) PCR=kPv

**Figure 3:**
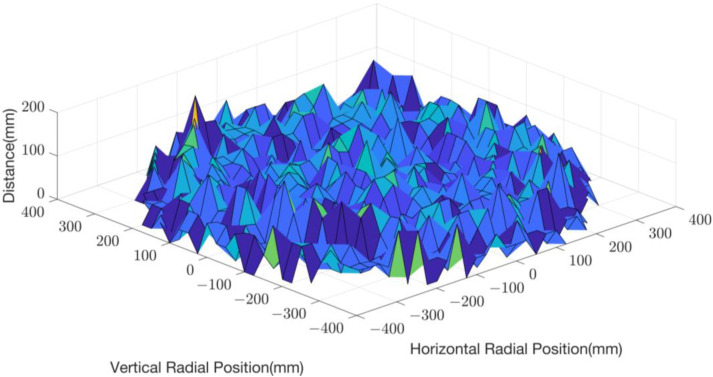
Surface map of the total scratch distance in each square unit of the example diamond (20 s).

Here, k is still an empirical constant based on the experimental data, which varies under different conditions. P is the downforce/pressure of the conditioner, and v is the relative velocity between the disk and the pad. Then, we can obtain the equation for the pad cut amount (*PCA*):

(12) PCA=kP∫vdt=kPs

where s represents the scratch distance. After we calculated the total scratch distance of the example diamond in Figure 2 in each square unit, we obtained the surface map of its total scratch distance in each square unit, as shown in Figure 3.

**Figure 4:**
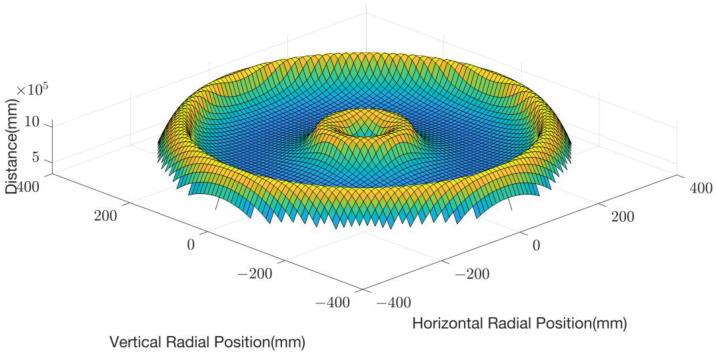
Surface map of the total trajectory distance (60 s) of all diamonds in each square unit under the sinusoidal sweep mode.

Under the sinusoidal sweep mode, we counted the results of all diamonds in each unit and obtain the real PCA pattern for the pad wear profile prediction, as shown in Figure 4.

## 3. Experimental Verification

**Figure 5:**
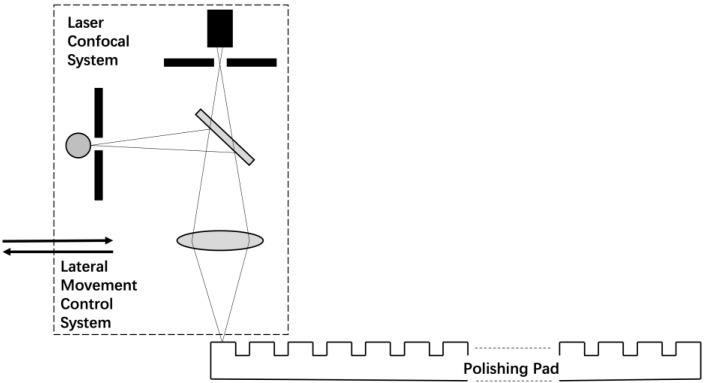
Schematic of the laser confocal system.

The verification experiment was conducted on a test platform (also manufactured by Hwatsing Co., Ltd., Tianjin, China) which has the same kinematic parameters as the industrial one. An online laser confocal system is integrated into this platform. It consists of an OLS5100 observing system (manufactured by OLYMPUS Co., Tokyo, Japan) and a lateral movement control system on the platform. A schematic of the system is shown in Figure 5. The OLS5100 system has a depth of focus of 10 mm.

**Figure 6:**
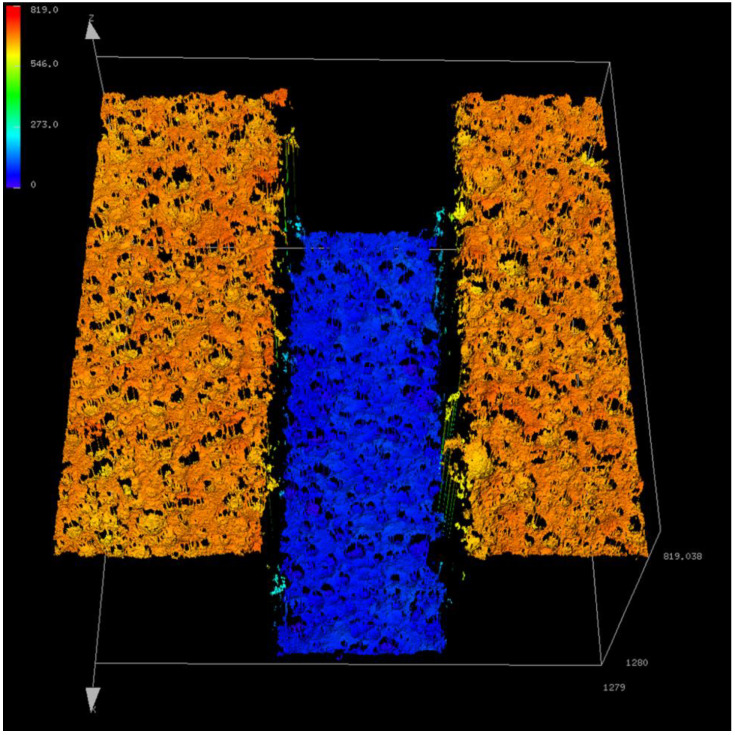
A 3D image of the pad surface generated by the OLS5100 system (1280 μm × 1280 μm).

**Figure 7:**
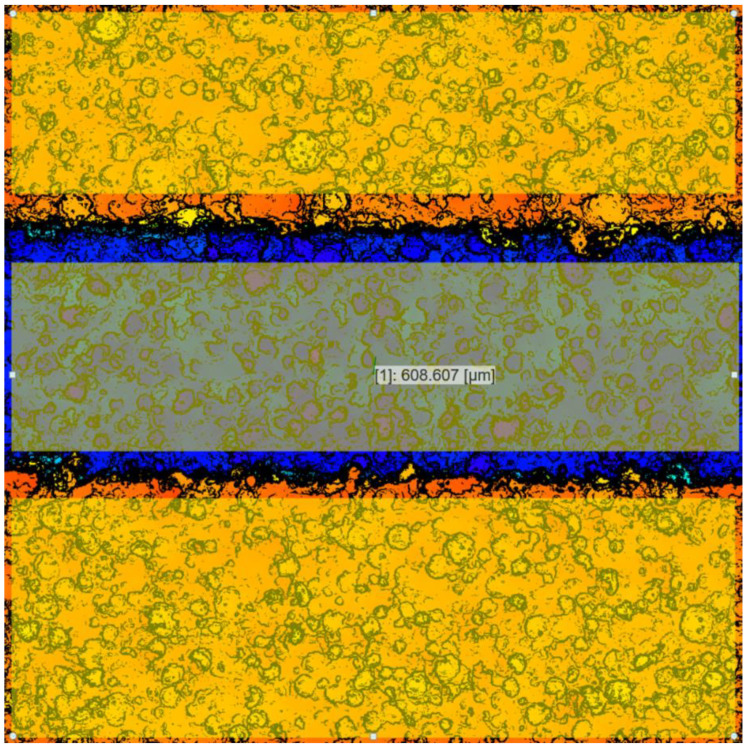
The 2D version image of Figure 6 and the analysis operation of the groove depth using the analytical software of the confocal system.

Figure 6 presents a 3D image of the pad surface generated by the OLS5100 system. Figure 7 shows the 2D version image of Figure 6 and the analysis operation of the groove depth using the analytical software of the confocal system (Analysis Application, Version 2.1.2.215). In the image, “608.607 μm” represents the difference in the average height between the middle quadrilateral region (groove bottom) and the other two quadrilateral regions (pad surface).

**Figure 8:**
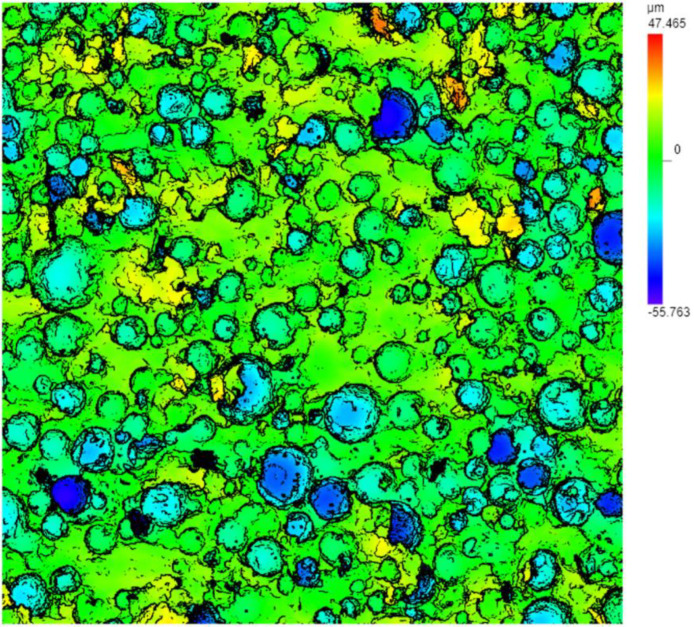
An image of the top pad surface generated by the OLS 5100 system (624 μm × 624 μm) showing the existence of the pores.

The polishing pad consists of three layers: top pad, sub pad, and the adhesive between them. The top pad of the polishing pads commonly used in industry is made of polyurethane, a polymer. In manufacturing, pores ranging from micrometers to hundreds of micrometers are added to the top pad, as shown in Figure 8, in order to adjust the hardness and other mechanical properties of the top pad and increase the feeding capacity of the polishing slurry and the removal capacity of waste materials. The pores can severely influence the average height calculation when a curved line passes them. Thus, the adoption of the line step height calculation needs a selection and processing of the height distribution curve. On the other hand, the adoption of the area step height calculation can significantly reduce the influence of the pores on the average height calculation of the surface and groove bottom baselines. A comparison between the groove heights of almost identical spots on the polishing pad before and after the break-in process leads to the PCR result during this process on that spot. The angles of the platen and positions of the lateral movement control system can be controlled almost precisely without removing the pad from the platen or touching it. Due to these online operations, the repeat precision can be lower than 2 μm.

The specific experimental conditions are listed in Table 1. The sinusoidal sweep mode was adopted in the verification experiment because it has more continuous and controllable motion during oscillation, and also the final result has a better recognizable pattern. The polishing pad and conditioner disk are both industrial-class products (pad manufactured by Hubei Dinghui Microelectronics Materials CO., Wuhan, Ltd., China; disk manufactured by QDH Semiconductor Co., Ltd., Singapore). The pad and disk combination is commonly seen in the industrial process. Other polyurethane-top-pad polishing pads and diamond disks can also be used to replace these chosen ones.

**Figure 9:**
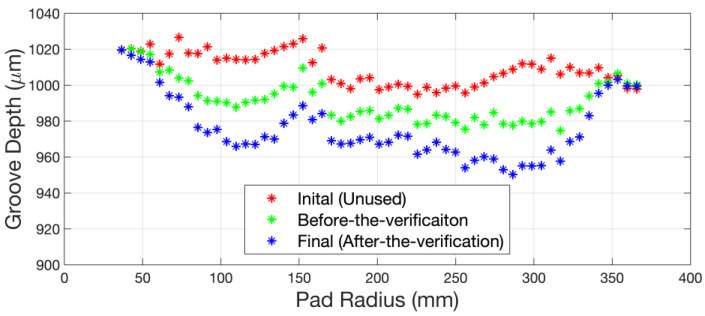
The initial (unused), pre-verification, and final (post-verification) groove depth profile of the polishing pad for this experiment.

**Figure 10:**
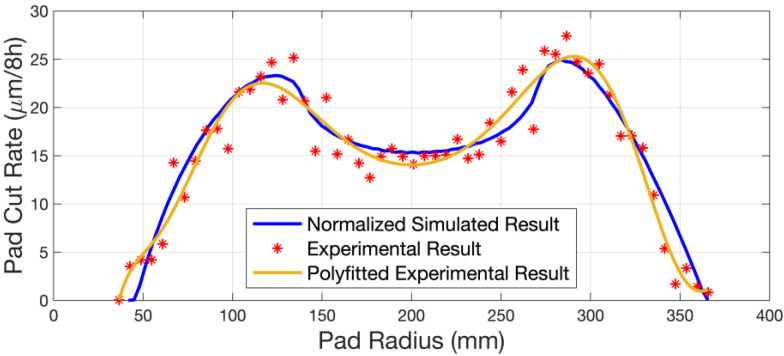
Comparison between the experimental and simulated results of the PCR under the sinusoidal sweep mode.

Figure 9 shows the initial (unused), pre-verification, and final (post-verification) groove depth profile of the polishing pad for this experiment. It should be noted that the initial (unused) polishing pad surface profile is not equal to the initial (unused) polishing pad groove depth profile. Figure 10 shows the comparison between the experimental and simulated results of the PCR under the sinusoidal sweep mode. The experimental results show a good approximation to the simulated ones, which verifies the effectiveness of the kinematic model that we constructed. Moreover, with the groove depth profile changing during conditioning, which means a change in the polishing pad surface profile, we can see that the PCR result is basically unaffected by the original surface profile of the polishing pad. Thus, the kinematic model is suitable for the prediction of the pad wear profile without considering the influence of mechanical parameters.

## 4. Results and Discussion

### 4.1. Influence of Different Kinematic Parameters on the PCR

**Figure 11:**
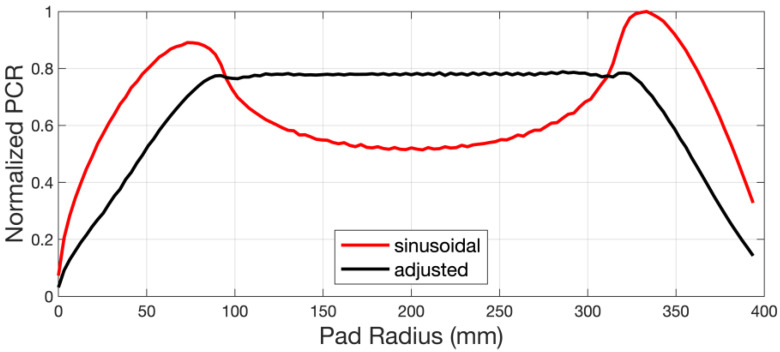
Simulated results referring to Table 3 (sweep range: radial 46~365 mm).

For feasible operation, the sweep mode cannot be fully defined by linear functions or trigonometric ones, it is usually divided into several partitions and the percentage of the dwelling time of the disk center in each partition is then set and later fitted into a polynomial function using a spline function. Table 2 lists the time settings of the sinusoidal sweep mode and the adjusted sweep mode. The adjusted sweep mode is for an optimized pad wear profile, as shown in Figure 11.

**Figure 12:**
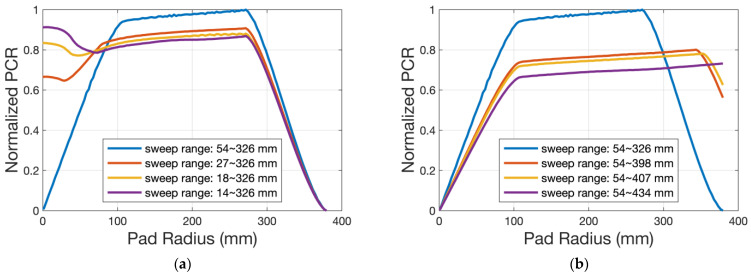
Simulated results under different sweep modes: (**a**) PCR pattern influenced by different start points; (**b**) PCR pattern influenced by different end points.

**Figure 13:**
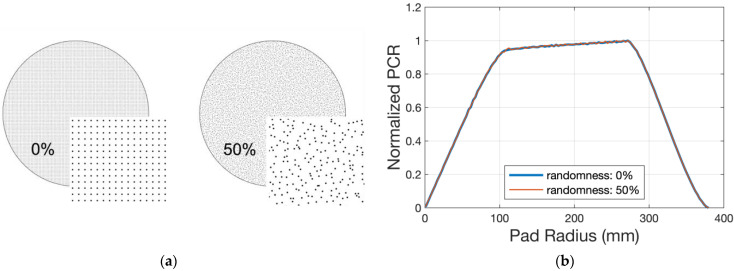
Simulated results under different diamond distribution randomness: (**a**) schematic of diamonds’ distribution under two kinds of randomness (0% as ordered arrangement and 50% as arrangement at maximum randomness); (**b**) simulated results referring to (**a**).

The PCR pattern is influenced mostly by the sweep mode and sweep range. Figure 12 presents the simulated results under different start and end point conditions. To keep the pad surface planar during polishing or only conditioning, we can change the sweep mode and range of the conditioner arm. On the other hand, within the scope of this kinematics model, the diamond distribution randomness on the disk surface has no effect on the PCR magnitude or pattern. Figure 13a shows the schematic of diamond distribution under two kinds of randomness (0% as an ordered arrangement, and 50% as the arrangement at maximum randomness). As shown in Figure 13b, the simulated results are similar.

### 4.2. Influence of the Pad Surface Unevenness on the Polishing Effects

**Figure 14:**
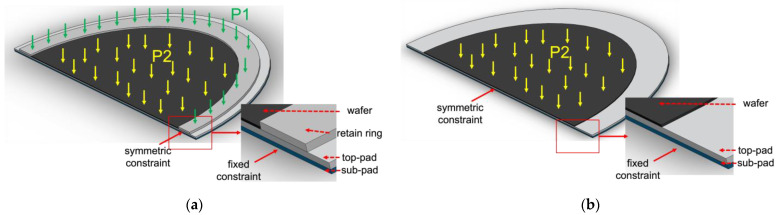
Schematics of the static model of the pad–wafer contact area: (**a**) with retaining ring; (**b**) without retaining ring.

Since there exists unevenness on the whole pad surface, we set up a static model to preliminarily investigate its influence on the pad–wafer contact stress. Figure 14 shows the schematic of the static model of the pad–wafer contact area with/without a retaining ring under common conditions. As mentioned above, the polishing pad and the wafer form an ultra-thin structure in the mechanical model due to its thickness–diameter quantitative relationship, which requires high computational power. Therefore, we ignored the existence of the pad’s and retaining ring’s groove, and those grids that are far away from the contact area of the polishing pad. As mentioned above, the polishing pad consists of three layers: top pad, sub pad, and the adhesive between them. As show in Figure 8, pores disperse in the top pad at a certain density. The adhesive and pores are both ignored in this simplified model. Meanwhile, due to the axial symmetry of the contact area between the polishing pad and the wafer along the radial direction of the polishing pad, only half of the contact area is taken. Symmetric constraints are applied on the surface of the cross sections of the polishing pad (including the top pad and the sub pad), wafer, and retaining ring. A fixed constraint is applied on the bottom of the sub pad. In the process of this mechanical modeling, all contact parts adopt the method of symbiotic nodes, which have the same strain and opposite stress.

**Figure 15:**
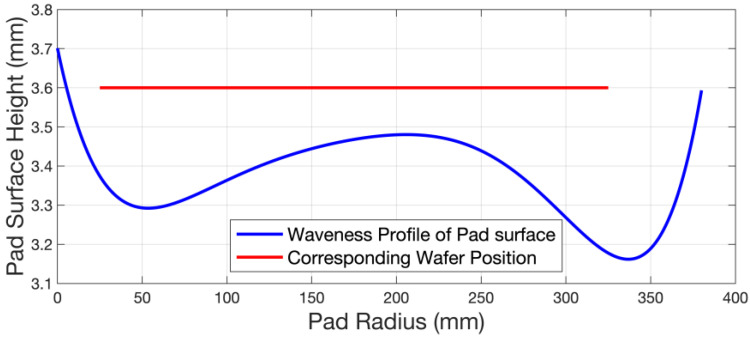
Pad surface profile and corresponding wafer position above it.

**Figure 16:**
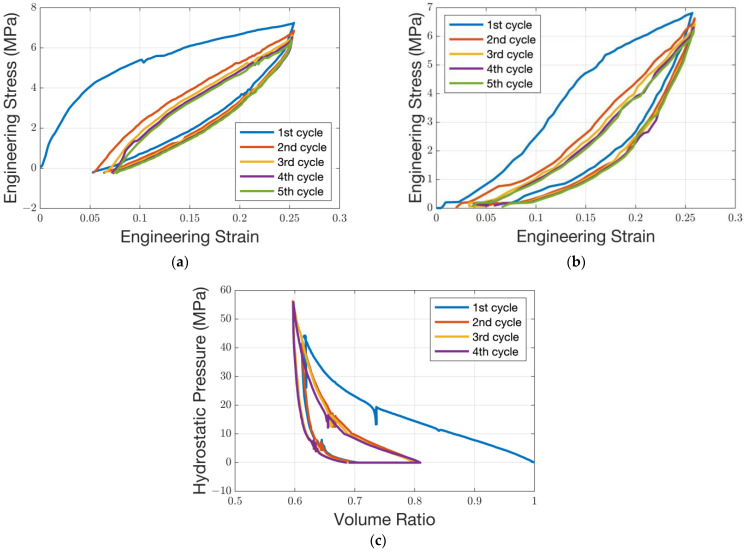
Mechanical properties of the top pad: (**a**) uniaxial test; (**b**) planar test; (**c**) volumetric test.

The static model was constructed using the commercial software ANSYS (Ansys, Inc., Version Build 21.1 UP20201109 (2021R1), Canonsburg, PA, U.S.). Table 3 lists the values of the parameters used in the static model. Figure 15 presents the pad surface profile, which can be obtained in real industry processes after a long marathon test, and the corresponding wafer position (symmetrical axis) above it. Since the top pad of the polishing pad is usually made of polyurethane, which is a super-elastic material, the deformation of the polishing pad under stress is very complex and cannot be characterized by only a few coefficients as the metallic materials. In order to obtain an intrinsic model of the polishing pad, a series of mechanical property tests are performed on a universal pressure testing machine (WDW-10M, Jinan Zhongluchang Testing Machine Manufacturing Co., Ltd, Jinan, China) at an ambient temperature of 70 ℃. This temperature setting is used because, during the CMP process, the friction between the ring and wafer and the polishing pad will generate a certain amount of heat. Figure 16a,b present the stress–strain curves of the uniaxial test and planar test under cyclic loading. Figure 16c presents the variation in the volume ratio and hydrostatic pressure. Its stress–strain relationship is often not a simple linear correlation. After several cycles, its curves gradually stabilize during these tests. These data can be directly input into ANSYS and further fitted by a Mooney–Rivlin model, and thus, the constitutive model of the top pad is derived. The fitting model can provide different responses for different stress deformation situations, rather than just a few rough parameters. As mentioned above, the retaining ring is a part of the polishing head. Usually, it is stuck to a stainless steel ring, which is fixed to the polishing head with bolts. The size of it in this model is consistent with an industrial one. However, the stainless steel ring and the other parts of the polishing head are simplified. The polishing head holds the wafer and presses it on the polishing pad.

**Figure 17:**
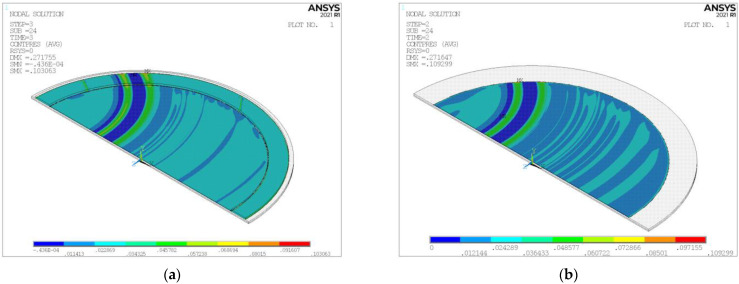
Static simulation results of pad–wafer contact stress: (**a**) with retaining ring; (**b**) without retaining ring.

**Figure 18:**
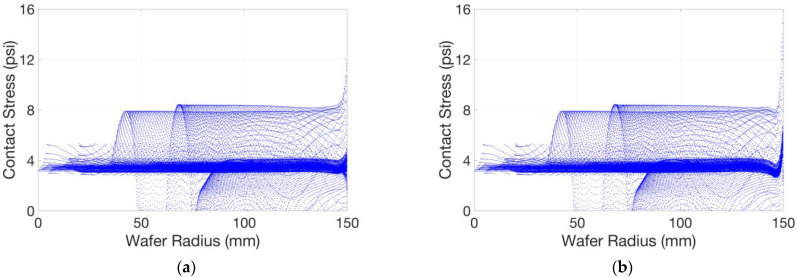
Scatter plots of stress distribution of the wafer surface along the radial direction: (**a**) with retaining ring; (**b**) without retaining ring.

**Figure 19:**
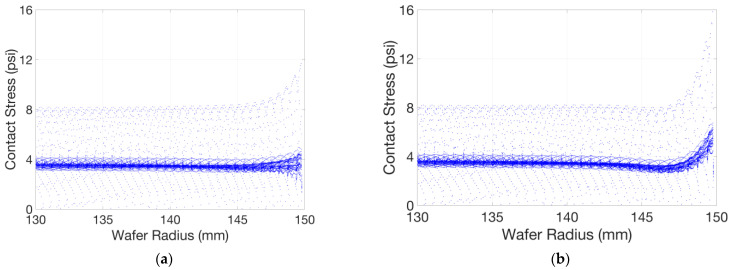
Scatter plots of stress distribution of the wafer surface along the radial direction at 130~150 mm: (**a**) with retaining ring; (**b**) without retaining ring.

Figure 17 shows the static simulation results of the pad–wafer contact stress, with or without the retaining ring. The pad–wafer contact status in this static model can be approximated as an instantaneous state in a dynamic model. We extracted the effective stress nodes on the wafer surface and drew scatter plots of the stress distribution of the wafer surface along the radial direction, as shown in Figure 18. Apart from the mainstream data and those below it, stress concentration occurs due to the pad unevenness, with a contact stress of some nodes in the central area that is two times that in the mainstream and a contact stress of some nodes in the edge area that is three (in (a)) or four times (in (b)) that in the mainstream. Stress concentration can increase the likelihood of defects such as scratches on the wafer surface, especially when the polishing slurry particles are involved. Observing the mainstream of the stress distribution, we can also find out that a smooth polishing pad is beneficial for a uniform stress distribution, as we imagined; and as shown in Figure 19, the pattern of the mainstream curve without the retaining ring first decreases in the edge area and then significantly warps upwards, and the fact that the stress concentration is more severe compared to that with the retaining ring indicates that a retaining ring helps to overcome the edge over-polishing effect, as stated in many other studies [12,13].

**Figure 20:**
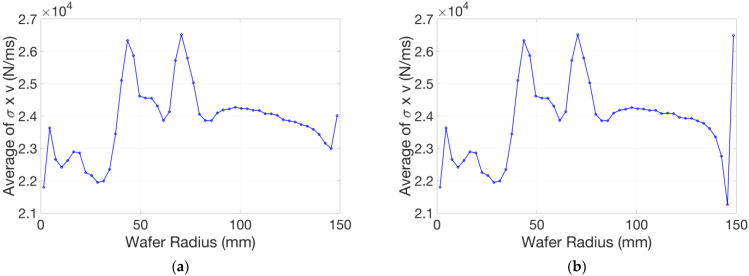
Circumferential average of the stress x relative velocity (σ×v) of the wafer surface along the radial direction: (**a**) with retaining ring; (**b**) without retaining ring.

According to the Preston equation, the MRR of an area is proportional to the relative velocity and the stress of that node. In the meanwhile, the multi-zone chamber inside the polishing head is concentric and the wafer keeps rotating inside the polishing head during polishing, especially in a dynamic model. Therefore, with the relative velocity [16] and stress of that node, we calculated the circumferential average of the stress x relative velocity (σ x v) of the wafer surface along the radial direction, as shown in Figure 20. During the calculation operation, referring to Figure 20, the node stress of the other half wafer, simplified when constructed, was also brought into consideration. Actually, the sum of the abscissa component of the relative velocity at two arbitrary symmetric points caused by the polishing pad rotation or the polishing head rotation is zero. Here, the Cartesian coordinate system was still fixed on the polishing pad, and the center of the polishing head was fixed on the horizontal axis eccentrically. The symmetry plane of these two arbitrary points is along the radial direction of the polishing pad. Meanwhile, the stress on these two points is equal. The oscillation speed of the polishing head, whose ordinate component is zero, was ignored in the calculation of the circumferential average in this static model; the reasons are as follows: firstly, the static model sets the corresponding wafer position above the polishing pad at a certain point. The standard oscillation mode of the polishing head is also sinusoidal, and its velocity at this point changes when the sweep range changes; secondly, it is smaller by an order of magnitude compared to the ordinate component of the relative velocity caused by the polishing pad rotation and the polishing head rotation together at most points. Those points where the oscillation velocity does matter are distributed near the vertical lines that are equally close to the centers of the polishing pad and the polishing head. Their influences can be easily weakened when we perform a circumferential average. Comparing these two calculated results, the positive effect of the retaining ring is re-acknowledged. They all reveal that the pad unevenness increases the difficulty of multi-zone pressure control.

## 5. Conclusions

We set up a kinematic model to predict the pad wear profile caused by only diamond disk conditioning and verified it. This model shows the influences of different kinematic parameters. To keep the pad surface planar during polishing or only conditioning, we can change the sweep mode and range of the conditioner arm. On the other hand, randomness of the diamond distribution has little influence on the result. This kinematic model can also investigate parameters such as disk size and diamond pitch. The kinematic model is suitable for the prediction of the pad wear profile without considering the influence of mechanical parameters.

Furthermore, based on the pad wear profile obtained from a real industrial process, we set up a static model to preliminarily investigate the influence of pad unevenness on the pad–wafer contact stress. The pad–wafer contact status in this static model can be approximated as an instantaneous state in a dynamic model. The model shows that the existence of a retaining ring helps improve the wafer edge profile and that the pad surface unevenness can cause stress concentration and increase the difficulty in multi-zone pressure control of the polishing head. The polishing slurry distribution on the polishing pad and the slurry particles are ignored in this simplified static model, as well as the pad grooves and pores. All these should be further discussed in the dynamic model construction, especially the slurry distribution [17].

## Figures and Tables

**Table 1:** Conditions of the verification experiment.

Condition	Status
Pad product type	DH3002
Pad total thickness	About 3.5 mm unused
Platen diameter	380 mm
Pad diameter	15.15 inches (385 mm) About 380 mm on the platen (effective)
Disk product type	OPTECH M1685
Disk diameter	108 mm 104.5 mm with diamonds on it (effective)
Average diamond pitch	430 μm
Average diamond width	150 μm
Average diamond protrusion	250 μm
Pad rotating speed	100 RPM
Disk rotating speed	73 RPM
Disk downforce	4 lbf
Conditioner arm sweep mode	Sinusoidal mode
Conditioner arm sweep speed	19 RPM
Conditioner arm sweep range	Radial 83~308 mm
Conditioning total time	8 h
Slurry	DI-water

**Table 2:** Time settings of 2 different 13-partition-control sweep modes.

Partition No.	1	2	3	4	5	6	7	8	9	10	11	12	13
Sinusoidal (%)	17.89	7.77	6.24	5.53	5.15	4.96	4.9	4.96	5.15	5.53	6.24	7.77	17.89
Adjusted (%)	8.47	7.24	7.29	7.33	7.37	7.42	7.46	7.5	7.55	7.59	7.63	7.72	9.42

**Table 3:** Parameters referring to the static model.

Parameter	Status
Pad taken region	Concentric with wafer, diameter: 370 mm
Elastic modulus of sub pad	3.9 MPa
Poisson’s ratio of sub pad	0.4
Wafer diameter	300 mm
Wafer thickness	775 μm
Elastic modulus of wafer	193 GPa
Poisson’s ratio of wafer	0.3
Retaining ring material	Polyphenylene Sulfide (PPS)
Retaining ring (RR) width	Diameter 301~348 mm
Retaining ring thickness	2.54 mm
Elastic modulus of retaining ring	210 GPa
Poisson’s ratio of retaining ring	0.3
Downforce P1	4.3 psi
Downforce P2	3.5 psi
Grid size	1 mm
Amount of grid	With RR: approximately 1.2 million Without RR: approximately 0.95 million

## Data Availability

The data supporting the reported results by the authors can be sent by e-mail.
